# Human immunodeficiency virus and oral microbiota: mutual influence on the establishment of a viral gingival reservoir in individuals under antiretroviral therapy

**DOI:** 10.3389/fcimb.2024.1364002

**Published:** 2024-04-10

**Authors:** Diana Estefania Ramos Peña, Sylvie Pillet, Alan Grupioni Lourenço, Bruno Pozzetto, Thomas Bourlet, Ana Carolina Fragoso Motta

**Affiliations:** ^1^ Department of Stomatology, School of Dentistry, University of São Paulo, São Paulo, SP, Brazil; ^2^ Team Mucosal Immunity and Pathogen Agents (GIMAP), Centre International de Recherche en Infectiologie (CIRI), Institut national de la santé et de la recherche médicale (INSERM) U1111, Ecole Nationale Supérieure de Lyon, Université de Lyon, Université de Saint-Etienne, Saint-Etienne, France; ^3^ Department of Infectious Agents and Hygiene, University-Hospital of Saint-Etienne, Saint-Etienne, France; ^4^ Department of Basic and Oral Biology, Ribeirão Preto School of Dentistry, University of São Paulo, São Paulo, Brazil; ^5^ Department of Stomatology, Public Health and Forensic Dentistry, Ribeirão Preto School of Dentistry, University of São Paulo, São Paulo, Brazil

**Keywords:** oral microbiota, microbiome, mycobiome, virome, HIV infection, reservoir, dysbiosis, periodontitis

## Abstract

The role of the oral microbiota in the overall health and in systemic diseases has gained more importance in the recent years, mainly due to the systemic effects that are mediated by the chronic inflammation caused by oral diseases, such as periodontitis, through the microbial communities of the mouth. The chronic infection by the human immunodeficiency virus (HIV) interacts at the tissue level (e.g. gut, genital tract, brain) to create reservoirs; the modulation of the gut microbiota by HIV infection is a good example of these interactions. The purpose of the present review is to assess the state of knowledge on the oral microbiota (microbiome, mycobiome and virome) of HIV-infected patients in comparison to that of HIV-negative individuals and to discuss the reciprocal influence of HIV infection and oral microbiota in patients with periodontitis on the potential establishment of a viral gingival reservoir. The influence of different clinical and biological parameters are reviewed including age, immune and viral status, potent antiretroviral therapies, smoking, infection of the airway and viral coinfections, all factors that can modulate the oral microbiota during HIV infection. The analysis of the literature proposed in this review indicates that the comparisons of the available studies are difficult due to their great heterogeneity. However, some important findings emerge: (i) the oral microbiota is less influenced than that of the gut during HIV infection, although some recurrent changes in the microbiome are identified in many studies; (ii) severe immunosuppression is correlated with altered microbiota and potent antiretroviral therapies correct partially these modifications; (iii) periodontitis constitutes a major factor of dysbiosis, which is exacerbated in HIV-infected patients; its pathogenesis can be described as a reciprocal reinforcement of the two conditions, where the local dysbiosis present in the periodontal pocket leads to inflammation, bacterial translocation and destruction of the supporting tissues, which in turn enhances an inflammatory environment that perpetuates the periodontitis cycle. With the objective of curing viral reservoirs of HIV-infected patients in the future years, it appears important to develop further researches aimed at defining whether the inflamed gingiva can serve of viral reservoir in HIV-infected patients with periodontitis.

## 1 Introduction

The oral microbiota, one of the more complex microbial communities in the human body, has become more understandable with the technological advances in culturomics (Lagier et al., 2018) and metagenomics (Costa and Weese, 2019; Liu et al., 2021). Its role in the overall health and in systemic diseases has gained more importance, mainly due to the systemic effects that are mediated by the chronic inflammation caused by oral diseases, and notably periodontitis, through the microbial communities of the mouth (Peng et al., 2022).

In healthy individuals, the oral microbiota is constituted by a variety of microorganisms living in community, notably through biofilms; the interaction between the communities is mainly based on mutualism and commensalism, maintaining a degree of homeostasis, also called eubiosis, which can vary in each person according to genetic and environmental conditions (Ptasiewicz et al., 2022). However, when alterations of the environment do occur, the interaction can become dominated by parasitism that is characterized by a disruption of homeostasis between microorganisms called dysbiosis, which results from the imbalanced interaction between the local microbiota and the host inflammatory response. This condition can lead to several oral diseases, including caries, periodontitis, and oral candidiasis (Hajishengallis and Chavakis, 2021; Ptasiewicz et al., 2022). In the presence of dysbiosis, the oral mucosa loses its impermeability to microorganisms, allowing the translocation of bacteria or their subproducts such as lipopolysaccharides (LPS) into the organism, without presence of bacteremia (Younas et al., 2019; Ptasiewicz et al., 2022). The resulting chronic low-grade inflammatory response, as the one found in periodontitis, can have implications in systemic chronic diseases, including cardiovascular diseases, diabetes, rheumatoid arthritis, inflammatory bowel disease or Alzheimer’s disease (Hajishengallis and Chavakis, 2021; Peng et al., 2022).

Regarding infectious diseases, the human immunodeficiency virus (HIV) infection –mainly linked to type 1 virus- has a great effect on the occurrence of opportunistic infections and cancers due to the chronic immunosuppression induced by the HIV viremia. By the end of 2022, there were 39 million people living with HIV (PLWH) worldwide, of these 29.8 million were receiving antiretroviral therapy (ART) (World Health Organization, 2023). It is estimated that approximately 50% of PLWH will present oral lesions at some point of the disease evolution, and this number goes up to 80% for the patients in the acquired immunodeficiency syndrome (AIDS) stage (Lomelí-Martínez et al., 2022). Several oral lesions in PLWH are related to bacterial, fungal or viral co-infections (for reviews see de Jesús-Campos et al., 1992; Ryder, 2000; Parveen et al., 2007; Lomelí-Martínez et al., 2022).

In the gut of PLWH, microbial dysbiosis contributes to the breakage of the intestinal immune barrier, leading to the translocation of pathogenic microbial products and a hyperactive inflammatory response resulting in CD4+ T cell exhaustion, suppression of the T regulatory (Treg) cell response and Th17 dysfunction (Brenchley et al., 2006, 2008; Mudd and Brenchley, 2016; Geng et al., 2020; Nganou-Makamdop et al., 2021; Shukla et al., 2021). Gut microbial dysbiosis and residual inflammation (local and systemic) present a feed-forward cycle that can be found even in long-term ART-treated patients, enabling the persistence of HIV infection due to the increase of the viral reservoir size (Koay et al., 2018; Coker et al., 2021; Ishizaka et al., 2021). In addition, different microbiota alterations have been described in the gut of patients under treatment with the nucleoside analog reverse-transcriptase inhibitor (NRTI) zidovudine, and/or the non-nucleoside reverse-transcriptase inhibitors (NNRTIs) efavirenz (Ray et al., 2021).

Similarly, alterations in the oral microbiota have been noticed in PLWH, yet not as widely studied as in the gut microbiota. Indeed, at the oral cavity level, the HIV-associated interactions between oral microbiota and mucosal immune cells may result in profound alterations of the microbiota, with some of these effects still being observed in well-controlled PLWH under ART, as described in the gut microbiota (Coker et al., 2021). Non-human primate models infected with simian immunodeficiency virus (SIV) bring additional information: the chronic inflammatory response observed in the mouth and plasma has been related to the presence of dysbiosis in the oral microbiota, suggesting a synergic interaction between SIV and dysbiosis (Giavedoni et al., 2013; Ocon et al., 2013). Although these animal models present an alternative for better controlling the environmental variables that affect microbiota studies, discrepancies can be observed within the different models and also with findings recorded in human beings (Brenchley & Ortiz, 2021).

Whereas a significative number of reviews of the literature have been dedicated to the relationship between gut microbiota and HIV infection, only few of them concern oral microbiota and are limited to a specific population (Starr et al., 2018) or to overall considerations (Moyes et al., 2016; Coker et al., 2021). The aim of the present review is to analyze the available literature on the different aspects of oral microbiota (bacteria, fungi and/or viruses) in PLWH, to compare the results with those of control subjects when tested in parallel, to investigate the reciprocal influence of periodontitis on HIV infection and oral microbiota, and to discuss the potential establishment of an HIV reservoir at the gingival level and its further consequences on the evolution of HIV disease. Different clinical and biological parameters that can alter the oral microbiota in the course of HIV infection are taken into consideration including age, immune and viral status, antiretroviral treatments, smoking, infection of the airway and viral coinfections.

## 2 Methods

An integrative review of the literature was conducted through an electronic search for studies in the MEDLINE/PubMed database, and Google Scholar for grey literature, without language restrictions, from 1996 onwards, which corresponds to the beginning of the ART era. Practically, the oldest studies are from 2007, in phase with the development and application of powerful molecular tools for investigating the various microbiota. The search strategy considered free text terms and controlled vocabulary related to “human immunodeficiency virus”, “HIV”, “HIV infection”, “HIV-infected patients”, “oral microbiota”, “oral microbiome”, “oral bacteria”, “oral mycobiome”, “oral fungi”, “oral candida”, “oral virome”, “oral human papillomavirus load”, “oral HPV load”, “oral Epstein-Barr virus load”, “oral EBV load”, “oral cytomegalovirus load”, “oral CMV load”, “oral herpes simplex virus load”, “oral HSV load”, “periodontitis” and “periodontal disease”. Additional references were identified from the bibliography of selected articles.

The inclusion criteria were observational, experimental, and quasi-experimental studies in human subjects that assessed the oral microbiota of PLWH and controls. As detailed below, some studies included subpopulations as young people, smokers or individuals with impaired lung function. A special attention was given to PLWH exhibiting periodontal disease. The exclusion criteria were studies that focused on a specific pathogen (except for *Candida* and a few viruses) and those that included only clinical considerations.

## 3 Technical considerations and definitions

The impact of HIV infection on the oral microbiota has been debated in the last decades, with diverse results. The main sampling of oral microbiota across the reviewed studies was obtained from saliva, oral rinse, supragingival or subgingival biofilm, and less frequently from swabs of the oral mucosae. The bacterial communities of these sampling sites are greatly affected by local clinical variables, including oral hygiene, smoking, presence of caries, periodontitis, and oral mucosal lesions and/or infections, but also systemic variables related to HIV infection (Beall et al., 2023). HIV infection can be considered an important variable when assessing the oral microbiota; however, it does not affect specific species and works concomitantly with other clinical variables (Beall et al., 2023). It is estimated that HIV infection alone accounts for 1.1% of the variance in the microbiota, but when in junction with other variables such as periodontitis, the effect on the bacterial communities could be magnified (Griffen et al., 2019).

For the assessment of microbiota, the terms diversity and relative abundance are generally used. Alpha diversity is defined as the number of species present in a local ecosystem, while beta diversity refers to the variety of species present in a habitat, a measure that depends on the comparison of species present in two or more locations (Whittaker, 1972). Thus, greater alpha diversity would represent a greater quantity of microorganisms present in a given site, whereas greater beta diversity would represent a greater number of different species, whatever the overall quantity of microorganisms present at this site. Relative abundance is a component of biodiversity: it refers to how common an OTU (Operational Taxonomic Unit) (species, genus, family, phylum …) is in relation to other OTUs located in the same community (Whittaker, 1972). The assessment of diversity and abundance of the microbiota is important for understanding whether the microbiota can be considered in a state of symbiosis/eubiosis (balanced microbiota) or dysbiosis (altered microbiota).

## 4 Assessment of the oral microbiome

### 4.1 General aspects on oral bacteria associated to HIV infection

From 2007 to 2023, 29 studies having evaluated the oral bacterial assessment in PLWH and corresponding to the inclusion criteria defined above were selected and analyzed. A summary of each of these studies is presented in **Table 1**.

**Table 1:** Presentation of the 29 studies selected for the analysis of the oral microbiome in people living with HIV (PLWH).

	Ref.	Population characteristics	ART status	Immune/viral status in HIV-infected patients	Type of sampling	Method of assessment	Main bacteriological finding
1	Aas et al., 2007	14 HIV-infected adult males: - 5 with gingivitis - 8 with periodontitis - 1 with linear gingival erythema	NA	10 with CD4+ T cell count >300/mm^3^ and viral load <2,000 cp/ml 4 with CD4+ T cell count <200/mm^3^ and viral load >20,000 cp/ml	Subgingival biofilm	16S rDNA gene amplification and sequencing	Classical periodontal pathogens were not detected in patients with periodontitis and were mostly replaced by opportunistic species.
2	Gonçalves et al., 2007	72 HIV-infected adults: - 37 with periodontitis - 35 without periodontitis 100 control adults: - 49 with periodontitis - 51 without periodontitis	72 PLWH under ART	72 with CD4+ T cell count <200/mm^3^ and detectable viral load	Subgingival biofilm	Checkerboard DNA-DNA hybridization method	Controls showed higher prevalence and level of most bacterial species than PLWH. 73% of the species were more frequently detected in the control group, regardless of the periodontal status. When the microbiota of PLWH and controls with and w/o periodontitis was compared, the highest prevalence and/or level of most bacterial species were detected in controls with periodontitis by comparison to other groups.
3	Silva-Boghossian et al., 2008	42 HIV-infected children 36 control children	38 PLWH under ART	10 children with AIDS 1 with moderate immunosuppression	Saliva	Checkerboard DNA-DNA hybridization method	Most of the tested species were more prevalent in control children than in the HIV-infected group, although only few species showed significant differences between groups. HIV-infected children presented significantly lower prevalence and level of several bacterial species in saliva. HIV-infected children under ART presented low prevalence of oral lesions.
4	Iwai et al., 2012	15 HIV-infected adults with acute pneumonia 5 control adults with ventilator-associated pneumonia	4 PLWH under ART	14 with low CD4+ T cell count and detectable viral load 1 with high CD4+ T cell count and undetectable viral load	Pool of tongue swab and oro-pharyngeal rinse	16S rRNA gene amplification and sequencing	A significantly greater number of taxa were detected in the airways of the PLWH than of controls. Different pathogenic processes occur in HIV-infected individuals and the susceptibility of this population to recurrent pneumonia may be due to the presence of a compositionally distinct and substantially more diverse airway microbiota.
5	Mukherjee et al., 2014	12 HIV-infected adults 12 control adults	8 PLWH under ART	11 with CD4+ T cell count >200 /mm^3^ and detectable viral load 1 with CD4+ T cell count <200 /mm^3^ and detectable viral load	Oral rinse	454 pyrosequencing	The bacteriome of PLWH was very close to that of controls.
6	Li et al., 2014	10 HIV-infected adults before and 6 months after ART initiation 10 control adults	10 PLWH initiated ART at baseline	At baseline: - mean CD4+ T cell count of 313/mm^3^ - viral load from 9.0 × 10^3^ to 7.1 × 10^5^ cp/ml	Saliva	Conventional culture Denaturing gradient gel electrophoresis and human oral microbe identification by microarray	PLWH had higher levels of total cultivable microbes, including oral streptococci, lactobacilli, *Streptococcus mutans*, and *Candida* spp, than controls. The prevalence of some bacterial genus was increased after ART, whereas that of *Aggregatibacter* was significantly decreased.
7	Hegde et al., 2014	50 HIV-infected adults 30 control adults	50 PLWH without ART	13 CD4+ T cell count >200/mm^3^ 37 CD4+ T cell count <200/mm^3^ Viral load NA	Oral rinse	Culture in 5% sheep blood agar	In the PLWH group, a shift in oral microflora with a reduction in the isolation of viridans streptococci and *Streptococcus pneumoniae* was found. The antibiotic sensitivity pattern in both groups showed sensitivity to the most commonly used antibiotics.
8	Goldberg et al., 2015	16 HIV-infected children 5 control children	NA	11 with CD4+ T cell count >200/mm^3^ and undetectable viral load	Supra-gingival biofilm Swabs of gingiva, tongue and oral mucosa Saliva	V1-V3 16S rRNA gene amplification and sequencing 454 pyrosequencing	No significant difference in bacterial microbiota was found between HIV status (negative, positive controlled or not), dental characteristics or between individual teeth.
9	Kistler et al., 2015	37 HIV-infected adults 37 control adults	37 PLWH under ART	Mean CD4+ T cell count: 510/mm^3^ Viral load NA	Supra-gingival biofilm Saliva	16S rRNA gene amplification and sequencing 454 pyrosequencing	The number of species detected was significantly lower in the saliva of PLWH than in that of controls. A significant difference in the bacterial content of saliva between PLWH and controls was found, but not in plaque. The oral microbiome of PLWH and controls was found to be similar overall.
10	Noguera-Julian et al., 2017	40 HIV-infected adults 10 control adults	39 PLWH under ART	Mean CD4+ T cell count by periodontal status: - mild/none: 578.2/mm^3^ - moderate: 362.7/mm^3^ - severe: 351.9/mm^3^ 8 with detectable viral load	Saliva Biofilm Swab of cheeks	V4 16S amplification and sequencing	HIV status and periodontitis severity showed a statistically significant impact on microbiome composition but only accounted for a combined 2% of variation. Altered immune markers in PLWH did not show association with the oral microbiome. Control samples showed higher richness measures only in the moderate periodontitis group.
11	Presti et al., 2018	35 HIV-infected adults	All 35 PLWH before and after 24 weeks of ART	Mean CD4+ T cell count: 326 /mm^3^ Mean viral load: 30,136 cp/ml	Saliva before and after 24 weeks of ART	V4 16S amplification and sequencing	Bacterial communities demonstrated considerable variability both within participants and between timepoints, although they became more similar across all participants following 24 weeks of ART. Alpha and beta diversities did not differ significantly between samples taken at baseline and after 24 weeks of ART therapy.
12	Mukherjee et al., 2018	48 HIV-infected smoker adults 24 HIV-infected non-smokers adults 24 control smoker adults	72 PLWH under ART	Mean CD4+ T cell count: - 838.87/mm^3^ in HIV-infected smokers - 808.83 /mm^3^ in HIV-infected non-smokers Viral load NA	Oral rinse	V4 16S amplification and sequencing	The bacteriome was widely dispersed, without any noticeable difference between groups. Richness of oral bacteriome was significantly lower in HIV-infected smokers than that in control smokers. Diversity at phylum level of HIV-infected non-smokers was significantly lower than that of HIV-infected or control smokers.
13	Starr et al., 2018	154 perinatally HIV-infected adolescents 100 HIV-exposed uninfected adolescents	NA	NA	Subgingival biofilm	V3-V4 16S amplification and sequencing	Species richness and alpha diversity differed little between the two groups. However, fewer oral “health”-associated bacterial taxa were found in HIV-infected than in exposed uninfected youth.
14	Gonçalves et al., 2019	27 HIV-infected children/teenagers 30 control children/teenagers	27 PLWH under ART	CD4+ T cell count range: 60–1803 /mm^3^ 15 with undetectable viral load	Saliva Biofilm of the tongue Pool of supra and subgingival biofilm	V1-V2 16S amplification and sequencing	A higher bacterial richness of supragingival and subgingival biofilm was observed in HIV-infected individuals compared with control children/teenagers. Globally, HIV-infected children/teenagers oral microbiome shows more complexity than that of controls of the same age.
15	Lewy et al., 2019	4 HIV-infected women >50 years old 4 HIV-infected women <35 years old 8 control women paired by age	3 HIV-infected women under ART	CD4+ T cell count range: - >50 years: 10-495/mm^3^ i - <35 years: 15-333/mm^3^ Viral load: - >50 years: 13000-270,000 cp/ml - <35 years: 140,000-1,400,000 cp/ml	Saliva	V3-v4 16S amplification and sequencing	HIV infection is associated with a shift toward an increased pathogenic footprint of the salivary microbiome. In both older and younger adult women, HIV infection was associated with salivary dysbiosis. Older age was associated with increased bacterial diversity in both PLWH and control women.
16	Griffen et al., 2019	252 HIV-infected adults 89 control adults	252 PLWH under ART	CD4+ T cell count range: 1-1661/mm^3^ Viral load range: 19–1.74 × 10^6^ cp/ml	Oral rinse	V1-V3 16S amplification and sequencing	If only HIV status was considered, the difference in oral microbial community composition between the PLWH and control groups was significant. However, several clinical factors such as caries, periodontal disease and age weighted more significantly in the observed differences. The influence of HIV/ART was statistically significant but smaller in magnitude than other clinical factors.
17	Annavajhala et al., 2020	52 HIV-infected post-menopausal women with periodontitis: - none/mild: 4 - moderate: 16 - severe: 22	52 HIV-infected women under ART	44 with CD4+ T cell count >200 /mm^3^ 43 with undetectable viral load	Saliva Subgingival biofilm Stools	V3-V4 16S amplification and sequencing	Bacterial alpha diversity in plaque, saliva and gut was associated with different immunological markers. Overall bacterial communities differed significantly with periodontal disease severity in saliva and plaque samples. Lipopolysaccharide-positive bacteria previously linked to inflammatory outcomes were enriched at oral sites in patients with severe periodontitis.
18	Coker et al., 2020	94 HIV-infected children (HI) 98 HIV-exposed uninfected children (HEU) 94 HIV-unexposed uninfected children (HUU)	90 HIV-infected children under ART	Mean CD4+ T cell count: 1,021 /mm^3^ Viral load NA	Saliva	V3-V4 16S amplification and sequencing	Perinatal HIV infection was significantly associated with community composition; however, the immune status had a stronger impact on bacterial profiles. Age-stratified associations of perinatal HIV exposure on community composition was observed, with HEU children differing from HUU children in early life but becoming more similar to HUU children with age. Regardless of age, HIV infection or exposure, low CD4+ levels persistently alter the oral microbiota during the developmental period. However, the effect of perinatal exposure without infection appears transient. When comparing salivary microbiota of HI and HEU children from HUU children, immunosuppression had a more pronounced effect on salivary bacterial composition.
19	Yang et al., 2020	75 HIV-infected adult men 93 control men All analyzed by lung function	72 HIV-infected men under ART	Mean CD4+ T cell count: 743 /mm^3^ 3 with detectable viral load	Saliva	V4 16S amplification and sequencing	Oral microbiome composition differed by HIV and smoking status. Alterations of oral microbial communities were observed in HIV-infected individuals with abnormal lung function. No significant association between the oral microbiome and lung function was found in control individuals. Among HIV-infected individuals, neither alpha nor beta diversity differed by ART, CD4+ T cell count, or HIV viral load; those who were actively smoking had reduced alpha diversity in saliva compared with nonsmokers. In PLWH, the oral microbiome may serve as an easily accessible marker of lung dysfunction.
20	Guo et al. 2021b	44 HIV-infected adult MSM -11 CDC stage 0 -10 CDC stage 1 -13 CDC stage 2 -10 CDC stage 3 (AIDS) 11 control adult MSM		Mean CD4+ T cell count: -Stage 0: 373.49/mm^3^ -Stage 1: 637.86/mm^3^ -Stage 2: 334.00/mm^3^ -Stage 3: 73.53/mm^3^ Mean viral load: -Stage 0: 4.28 log_10_ -Stage 1: 3.80 log_10_ -Stage 2: 4.23 log_10_ -Stage 3: 4.66 log_10_	Saliva	V3-V4 16S amplification and sequencing	HIV-infected patients presented significantly greater alpha-diversity in the microbial composition compared to controls, except at AIDS stage. *Porphyromonas* was significantly less abundant in PLWH than in controls. Bacterial abundance increased during the acute HIV infection phase. In AIDS patients, partial inhibition of some bacteria was observed.
21	Imahashi et al., 2021	20 HIV-infected adult men 8 control adult men	20 HIV-infected men under ART - 6 NRTI - 9 NNRTI - 5 PI	Mean CD4+ T cell count: - NRTI: 288/mm^3^ - NNRTI: 526/mm^3^ - PI: 520/mm^3^ Viral load NA	Saliva Stools	V3-V4 16S amplification and sequencing	ART, especially NRTI-based ART, has remarkable impacts on fecal microbial diversity with decreased α-diversity and increased beta diversity over time. In contrast, dynamic diversity changes in the salivary microbiome were not observed. This suggests that ART has more suppressive impacts on microbiota composition and diversity in the gut than in the mouth, which potentially causes intestinal dysbiosis in PLWH but not in the oral cavity.
22	Li et al., 2021	15 acute HIV-infected adult MSM 15 chronic HIV-infected adult MSM 15 control adult MSM	30 HIV-infected men w/o ART	Mean CD4+ T cell count: - acute HIV: 397.4/mm^3^ - chronic HIV: 486.5 /mm^3^ Mean viral load (cp/ml): - acute HIV: 56407 - chronic HIV: 36592	Throat swab before and 12 weeks after ART initiation	V4-V5 16S amplification and sequencing	Microbiome diversity was significantly decreased in patients with acute and chronic HIV infections compared with controls before ART and the significant difference remained at 12 weeks after ART initiation.
23	Perez Rosero et al., 2021	62 HIV-infected adults 43 control adults	51 PLWH under ART	11 with low CD4+ T cell count < 200 /mm^3^ 40 with high CD4+ T cell count >200 /mm^3^ Viral load NA	Oral rinse Saliva	V3-V4 16S amplification and sequencing	The saliva samples from PLWH harbored significantly richer bacterial communities compared to the saliva samples from controls. The core oral microbiome was distinguishable between HIV-infected individuals on ART compared to the control group.
24	Xie et al., 2021	30 HIV-infected immunological responder (IR) adults 34 HIV-infected immunological non-responder (INR) adults	64 PLWH under ART	CD4+ T cell count: - IR: >500 /mm^3^ -INR: <200 /mm^3^ Viral load NA	Saliva	V3-V4 16S amplification and sequencing	The IR and INR groups presented similar salivary bacterial richness and diversity. The overall salivary microbiota structure was similar in the IR and INR groups, while there were some taxonomic differences in the salivary bacterial composition. Notably, the genus *Saccharimonas* could be considered in the future as a screening biomarker for the immune response in HIV-infected individuals.
25	Cao et al., 2022	454 pyrosequencing (PS): 5 AIDS adults before ART 5 AIDS adults after ART. 5 control adults RT-qPCR: 64 AIDS adults before ART 62 AIDS adults after ART 78 control adults	454 PS: 5 PLWH under ART RT-qPCR: 62 PLWH under ART	AIDS patients CD4+ T cell count NA Viral load NA	Saliva	V3-v5 16S amplification and 454 pyrosequencing RT-qPCR	Salivary microbiota was increased in PLWH compared to controls. A large number of pathogens were detected in the saliva of AIDS patients. ART therapy reduced the salivary microbiota diversity in AIDS patients.
26	Ramos Peña et al., 2022	18 HIV-infected adults with periodontitis 14 control adults with periodontitis	18 PLWH under ART	10 with CD4+ T cell count <500 /mm^3^ and undetectable viral load 8 with CD4+ T cell count <500 /mm^3^ and detectable viral load	Subgingival biofilm before and after periodontal treatment	V3-V4 16S amplification and sequencing	A low abundance of periodontopathogenic bacteria was observed; the periodontal treatment induced shifts in the subgingival biofilm of PLWH, leading to a microbiota similar to that of controls. Different subgingival microbiota profiles were identified. A less diverse microbiota was found in PLWH.
27	Beall et al., 2023	257 HIV-infected adults 93 control HIV-high risk adults	71 PLWH w/o ART	Mean CD4+ T cell count: 426 /mm^3^ Mean viral load: 43685 cp/ml	Oral rinse, before and after treatment for those under ART	V1-V3 16S amplification and sequencing	The oral bacteriome revealed significant differences between the 2 groups, contributed by several clinical variables including gingivitis, the presence of *Candida* spp, current cigarette smoking, age, periodontal disease, antibiotic therapy, and HIV status. These influences accounted for ~14% of the variance in the oral bacteriome, whereas HIV status accounted for only 1.1% of the identified variance. The independent effects of HIV status and ART therapy on the oral microbiome are significant and similar to those of the clinical variables but collectively modest.
28	Kuhn et al., 2023	477 HIV-infected children 123 control children	477 HIV-infected children under ART	Mean CD4+ T cell count: 927 /mm^3^ Viral load: - 431 undetectable - 44 detectable	Pooled swabs of tongue, palate, mucosa, and saliva	V4 16S amplification and sequencing	HIV-infected children had lower alpha diversity than controls and this association was not attenuated by earlier ART initiation. Shifts in genus-level taxa abundances in HIV-infected children relative to controls were most marked in those treated by lopinavir/ritonavir than in those receiving efavirenz. Oral bacterial diversity was consistently lower among HIV-infected children compared with controls regardless of pre-ART viral load or CD4+ cell count. A distinct profile of less diverse oral bacterial taxa was observed in HIV-infected children on ART compared with controls suggesting modulation of microbiota in the mouth by HIV and/or its treatments.
29	Meng et al., 2023	5 HIV-infected adults 12 adult controls	5 PLWH under ART	NA	Saliva Different locations in gut from colon endoscopy	V4 16S amplification and sequencing	By contrast to gut, the oral microbiome is not altered n PLWH. Salivary samples from PLWH did not cluster apart from salivary samples from control individuals. Oral samples from PLWH were not significantly different from control individuals in biofilm-forming bacteria, pathogenic bacteria, Gram-positive, or Gram-negative bacteria.

ART, antiretroviral therapy; CDC, Centers for Disease Control and Prevention; cps/ml, copies per ml; MSM, men who have sex with men; NA, not available; PLWH, people living with HIV; NNRTI nonnucleoside/nucleotide reverse transcriptase inhibitor; NRTI, nucleoside/nucleotide reverse transcriptase inhibitor; PI, protease inhibitor; PS, pyrosequencing; Ref., reference; w/o, without.

In the overall composition of oral microbiota, studies have pointed out similar microbiota between PLWH and controls, regarding richness, evenness, composition, or predicted functions (Mukherjee et al., 2014; Imahashi et al., 2021; Meng et al., 2023). However, when analyzing in more detail the bacterial diversity of the oral cavity, several differences can be noticed in PLWH with reference to controls. The search for understanding these differences in PLWH has been based on significant results found in the gut microbiota in HIV infection. Beta diversity of colon samples present significant differences between PLWH and controls, pointing out an association between HIV infection and gut dysbiosis. However, these significant changes have not been found in the oral microbiota when the only variable taken into consideration is HIV infection (Meng et al., 2023).

Regarding the impact of HIV-infection in the bacterial diversity of oral microbiota, conflicting results are found in the literature: while a few studies showed no significant difference between PLWH and controls (Mukherjee et al., 2014; Goldberg et al., 2015; Gonçalves et al., 2019; Meng et al., 2023), other studies found significant differences between both groups (Gonçalves et al., 2007; Silva-Boghossian et al., 2008; Iwai et al., 2012; Hegde et al., 2014; Li et al., 2014; Kistler et al., 2015; Noguera-Julian et al., 2017; Presti et al., 2018; Griffen et al., 2019; Lewy et al., 2019; Coker et al., 2020; Yang et al., 2020; Perez Rosero et al., 2021; Guo et al., 2021b; Cao et al., 2022; Ramos Peña et al., 2022; Beall et al., 2023; Kuhn et al., 2023).

In saliva samples and oral rinses, the main differences found between PLWH and controls were related to bacterial composition (Kistler et al., 2015; Presti et al., 2018; Yang et al., 2020; Perez Rosero et al., 2021), alpha and beta diversity (Li et al., 2014; Noguera-Julian et al., 2017; Mukherjee et al., 2018; Gonçalves et al., 2019; Annavajhala et al., 2020; Li et al., 2021; Guo et al., 2021b; Kuhn et al., 2023), whereas some of them reported differences in prevalence or abundance of specific genera and species (Silva-Boghossian et al., 2008; Hegde et al., 2014). These differences have been attributed mainly to the presence of HIV-infection and ART regimens.

### 4.2 Findings related to bacterial genera and species mostly found in HIV infection

**Figure 1:**
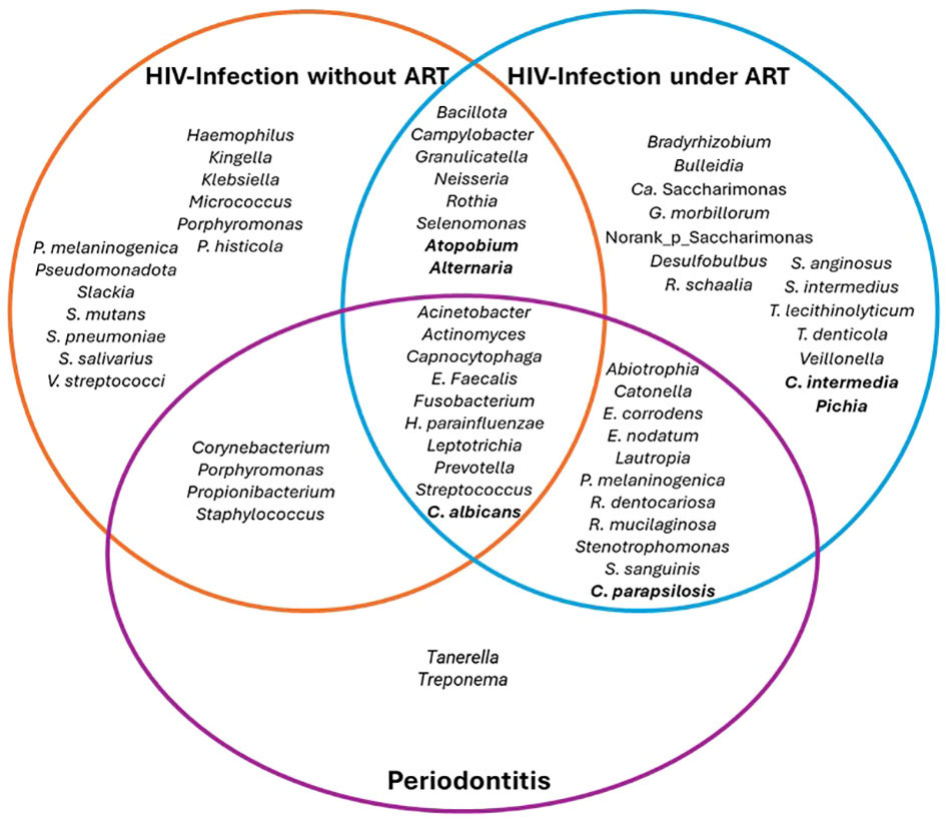
Main bacterial genera or species that were detected in HIV infection according to presence of ART and periodontitis. *Ca*: *Candidatus.*.

Differences in the overall bacterial population have been found in the oral microbiota of PLWH. The reported results regarding bacterial taxonomy in PLWH differed according to the type of samples, being saliva, biofilm, oral rinses and mucosal swabs, the most common methods used to assess the oral microbiota. The main taxonomical differences found in PLWH have been related to increased abundance of genera such as *Campylobacter*, *Granulicatella, Neisseria*, *Fusobacterium*, and *Selenomonas* (Li et al., 2014, 2021; Guo et al., 2021b; Cao et al., 2022), and decreased abundance of *Actinomyces, Lactobacillus, Peptostreptococcus* and *Treponema* (Gonçalves et al., 2007; Silva-Boghossian et al., 2008; Annavajhala et al., 2020; Ramos Peña et al., 2022). On the other hand, an association with increased abundance of *Lactobacillus*, *Lautropia* and *Bacteroides* has been reported in controls (Li et al., 2014; Imahashi et al., 2021; Li et al., 2021) (**Table 2** and **Figure 1**).

**Table 2:** Main taxa found increased/decreased in people living with HIV (PLWH) by comparison to HIV-negative controls by study (fungi in bold characters).

	Reference	Population characteristics	Type of sampling	Taxa in HIV-infected subjects by reference to HIV-negative ones	
				Increased	Decreased
1	Gonçalves et al., 2007	72 HIV-infected adults: - 37 with periodontitis - 35 without periodontitis 100 control adults: - 49 with periodontitis - 51 without periodontitis	Subgingival biofilm	Without periodontitis: *Enterococcus faecalis*	Without periodontitis: *Actinomyces naeslundii* *Actinomyces viscosus* *Aggregatibacter* *actinomycetemcomitans* *Capnocytophaga gingivalis* *Eubacterium nodatum* *Fusobacterium nucleatum* *Gemella morbillorum* *Porphyromonas gingivalis* *Prevotella intermedia* *Prevotella melaninogenica* *Prevotella nigrescens* *Selenomonas* *Streptococcus* *Streptococcus intermedius* *Tannerella forsythensis*
				Periodontitis: *Acinetobacter baumannii* *Capnocytophaga gingivalis* *Eikenella corrodens* *Enterococcus faecalis* *Eubacterium nodatum* *Propionibacterium acnes* *Streptococcus sanguinis*	Periodontitis: *Actinomyces naeslundii* *Capnocytophaga gingivalis* *Escherichia coli* *Eubacterium nodatum* *Fusobacterium nucleatum* *Gemella morbillorum* *Prevotella nigrescens* *Streptococcus mitis*
2	Silva-Boghossian et al., 2008	42 HIV-infected children 36 control children	Saliva	*Enterococcus faecalis* *Fusobacterium periodontium* *Gemella morbillorum* *Streptococcus intermedius* *Streptococcus anginosus* *Treponema denticola*	*Actinomyces gerencseriae* *Actinomyces meyeri* *Bacillus cereus* *Capnocytophaga gingivalis* *Enterococcus faecalis* *Eubacterium nodatum*, *Fusobacterium nucleatum* *Pseudomonas aeruginosa* *Prevotella intermedia* *Peptostreptococcus micros* *Staphylococcus aureus* *Streptococcus constellatus* *Selenomonas noxia* *Tannerella forsythia*
3	Mukherjee et al., 2014	12 HIV-infected adults 12 control adults	Oral rinse	*Capnocytophaga* *Prevotella* *Rothia* *Streptococcus* *Alternaria* *Epicoccum* *Candida intermedia* *Candida albicans* ***Pichia***	*Aggregatibacter* *Fusobacterium* *Prevotella* *Streptococcus* *Candida sake* *Candida albicans* *Fusarium*
4	Li et al., 2014	10 HIV-infected adults before and 6 months after ART initiation 10 control adults	Saliva	*Actinomyces* *Campylobacter* *Capnocytophaga* *Fusobacterium* *Granulicatella* *Lactobacilli* *Prevotella* *Selenomonas* *Streptococci* *Streptococcus mutans* ***Atopobium*** ***Candida* spp** *After ART:* *Actinomyces* *Campylobacter* *Capnocytophaga* *Fusobacterium* *Granulicatella* *Prevotella* *Selenomonas* *Atopobium*	*Aggregatibacter Capnocytophaga* *Atopobium* *Kingella* *Lactobacillus* *Peptostreptococcaceae* *Porphyromonas* *Slackia*
5	Hegde et al., 2014	50 HIV-infected adults 30 control adults	Oral rinse	*Micrococcus spp* *Viridans streptococci* *Acinetobacter* *Klebsiella spp* *Streptococcus pneumoniae*	N/D
6	Kistler et al., 2015	37 HIV-infected adults 37 control adults	Saliva	*Haemophilus parainfluenzae*	*Streptococcus mitis* *Streptococcus pneumoniae*
			Supra-gingival biofilm	No significant differences were observed between the biofilm bacterial communities of HIV-infected and HIV-negative subjects	
7	Noguera-Julian et al., 2017	40 HIV-infected adults 10 control adults	Saliva Biofilm Swab of cheeks	*Abiotrophia* *Kingella* *Neisseria* *Pseudomonadota* Periodontitis: *Abiotrophia* *Pasteurellaceae* *Rothia* *Treponema*	*Leptotrichia* *Selenomonas* Severe periodontitis: *Streptococcus*
8	Presti et al., 2018	35 HIV-infected adults	Saliva before and after 24 weeks of ART	Before ART: *Porphyromonadaceae* *Bacteroidota* *Bacillota* *Pseudomonadota* *H. parainfluenzae* After ART: *T. lecithinolyticum* After ART the dominant phyla in saliva remained similar to that found at baseline	Before ART: *Fusobacteria* *Spirochaetes* *Actinobacteria* *Ternericutes*
9	Mukherjee et al., 2018*	48 HIV-infected smoker adults 24 HIV-infected non-smokers adults 24 control smoker adults	Oral rinse	Non-Smokers: *Pelomonas* *Pseudomonadota* *Pseudomonas pseudoalcaligenes*	Non-smokers: *Bacillota*
				Smokers: *Chrytridiomycota* *Granulicatella* *Lactobacillus* *Veillonella* *Enhydrobacter* *Streptococcaceae* *Comamonadaceae* *Cladosporium* *Nakaseomyces* *Scleroderma* *Rhodotorula* ***Facklamia*** *Candida glabrata* ***Candida dubliniensis*** *Scleroderma*	Smokers: *Neisseria* *Pelomonas puraquae* *Pelomonas* *Chrytridiomycota* *Stemphylium solani* *Debaryomyces hansenii* *Olpidium brassicae*
10	Starr et al., 2018	154 perinatally HIV-infected adolescents 100 HIV-exposed uninfected adolescents	Subgingival biofilm	*Acinetobacter* *Leptotrichia sp.* *Streptococcus sp.* *Tanerella*	*Abiotrophia defectiva* *Actinobaculum* *Aggregatibacter actinomycetemcomitans* *Bacteroidales* *Corynebacterium* *Filifactor alocis* *Haemophilus* *Leptotrichia Shahii* *Leptotrichia wadei* *Porphyromonas endodontalis* *Prevotella histicola* *Prevotella melaninogenica* *Prevotella nigrescens* *Streptococcus mutans* *Tannerella forsythia* *Treponema genus*
11	Gonçalves et al., 2019	27 HIV-infected children/teenagers 30 control children/teenagers	Saliva	*Prevotella*	*Fusobacterium* *Leptotrichia* *Neisseria* *Pseudomonadota*
			Biofilm of the tongue	*Nesisseria* *Leptotrichia*	*Prevotella* *Veillonella*
			Supragingival biofilm	Bacillota *Streptococcus*	Fusobacteria *Proteobacteria*
			Subgingival biofilm	*Prevotella* *Veillonella*	N/D
12	Griffen et al., 2019	252 HIV-infected adults 89 control adults	Oral rinse	No species was identified as significantly differentially abundant between the HIV positive and negative groups	
13	Annavajhala et al., 2020**	52 HIV-infected post-menopausal women with periodontitis: - none/mild: 4 - moderate: 16 - severe: 22	Saliva	Severe periodontitis: *Fusobacterium* *Prevotella melaninogenica* *Rothia mucilaginosa* *Candida albicans* *Candida parapsilosis*	Severe periodontitis: Megasphaera ***Candida dubliniensis*** ***Exserohilum turcicum*** ***Guehomyces pullalans*** ***Debaryomyces hansenii***
			Subgingival biofilm	Severe periodontitis: *Catonella* *Fusobacterium* *Haemophilus parainfluenzae* *Lachnospiraceae* *Lautropia* *Leptotrichia* *Prevotella* *Rothia dentocariosa* *Streptococcaceae* *Streptococcus* *Veillonella dispar* ***Filobasidiales***	Severe periodontitis: *Streptococcus* *Actinomyces* *Granulicatella* *Veillonella dispar* ***Saccharomyces cerevisiae***
14	Coker et al., 2020	94 HIV-infected children (HI) 98 HIV-exposed uninfected children (HEU) 94 HIV-unexposed uninfected children (HUU)	Saliva	*Corynebacterium diphtheriae* Low CD4+ T cell count: *Actinomyces* *Porphyromonas pasteria* *Prevotella nanceiensis* *Rothia mucilaginosa* Without ART: *Actinomyces* *Leptotrichia* *Neisseria* *Streptococcus salivarius*	*Actinomyces* *Haemophilus* *Leptotrichia* *Neisseria subflava* *Peptidiphaga gingivicola* *Streptococcus mitis* *Streptococcus mutans*
15	Yang et al., 2020	75 HIV-infected adult men 93 control men	Saliva	*Rothia* *Streptococcus* *Veillonella* Smokers: *Lactobacillus* *Streptococcus* *Veillonella* Abnormal lung function: *Lactobacillus* *Streptococcus* *Veillonella*	*Neisseria* Smokers: *Neisseria* *Prevotella* *Pasteurellaceae* *Fusobacterium*
16	Guo et al. 2021b	44 HIV-infected adult MSM -11 CDC stage 0 -10 CDC stage 1 -13 CDC stage 2 -10 CDC stage 3 11 control adult MSM	Saliva	Stage 0: *Veillonella*, *Fusobacterium* *Alloprevotella* *Haemophilus* Stage 1: *Megasphaera* *Corynebacterium* Stage 2: *Veillonella* *Alloprevotella* *Megasphaera* *Aggregatibacters* *Selenomonas_3* *Campylobacter* Stage 3 *: Prevotella_6* *Campylobacter* *Shuttleworthia* *Dialister* *Solobacterium* *norank_f_* *Saccharimonadaceae*	Stage 0: *Porphyromonas* *Rothia* Stage 1: *Porphyromonas* *Lactobacillus* Stage 2: *Porphyromonas* *Prevotella* Stage 3: *Porphyromonas* *Rothia* *Haemophilus* *Prevotella_2* *Capnocytophaga* *Fusobacterium* *Leptotrichia*
17	Li et al., 2021	15 acute HIV-infected adult MSM 15 chronic HIV-infected adult MSM 15 control adult MSM	Throat swab before and 12 weeks after ART initiation	Before ART: *Actinomyces* *Bacteroidales* *Haemophilus* *Neisseria* *Prevotella histicola* *Prevotella melaninogenica* *Prevotellaceae* *Rothia* *Ruminococcaceae* *Streptococcus* After ART: *Bradyrhizobium*	Before ART: *Actinobacillus* *Alloprevotella* *Fusobacterium* *Haemophilus* *Oribacterium* *Veillonella* Low CD4+ T cell count: *Actinomyces* *Haemophilus* *Rothia* *Ruminococcaceae*
18	Perez Rosero et al., 2021	62 HIV-infected adults 43 control adults	Saliva	*Spirochaeta* (*Treponema, Treponema amylovorum*, and *Treponema azotonutricum*) *Bacteroidota (Prevotella and Elizabethkingia)* *Bacillota (Bacillaceae, Lactobacillales)* *Saccharibacteria*	*Helicobacter* sp
19	Xie et al., 2021	30 HIV-infected immunological responder adults 34 HIV-infected immunological non-responder adults	Saliva	Immunological non-responders: *Selenomonas* Immunological Responders: *Candidatus_Saccharimonas* *norank_p_Saccharimonas, Desulfobulbus*	Immunological Responders: *Selenomonas*
20	Cao et al., 2022	454 pyrosequencing: 5 healthy controls, 5 HAART processed AIDS patients 5 HAART unprocessed AIDS patients. RT-qPCR: 64 Before treatment of HAART-processed AIDS patients 62 After treatment of HAART-processed AIDS patients 78 healthy controls	Saliva	454 pyrosequencing after ART: *Campylobacter* *Capnocytophaga* *Fusobacterium* *Granulicatella* *Prevotella* *Selenomonas*	454 pyrosequencing: *Atopobium* *Capnocytophaga* *Kingella* *Lactobacillus* *Peptostreptococcaceae* *Porphyromonas* *Slackia*
				RT-qPCR after ART: *Atopobium* *Capnocytophaga* *Kingella* *Lactobacillus* *Peptostreptococcaceae* *Porphyromonas* *Slackia*	RT-qPCR before ART: *Atopobium* *Capnocytophaga* *Kingella* *Lactobacillus* *Peptostreptococcaceae* *Porphyromonas* *Slackia*
21	Ramos Peña et al., 2022	18 HIV-infected adults with periodontitis 14 control adults with periodontitis	Subgingival biofilm before and after periodontal treatment	*Fusobacterium* *Prevotella* *Stenotrophomonas* *Streptococcus* *Veillonella* Low CD4+ T cell count: *Streptococcus* *Stenotrophomonas* *Fusobacterium* *Veillonella* After periodontal treatment: *Prevotella*	*Defluviitaleaceae* *Filifactor* *Haemophilus* *Peptostreptococcus* *Porphyromonas* *Treponema* After periodontal treatment: *Actinomyces* *Anaeroglobus* *Stenotrophomonas*
22	Beall et al., 2023	257 HIV-infected adults 93 control HIV-high risk adults	Oral rinse, before and after treatment for those under ART	N/D	*Fusobacterium nucleatum* *Porphyromonas pasteri* *Prevotella denticola* *Streptococcus salivarius* *Candida dubliniensis*
23	Kuhn et al., 2023	477 HIV-infected children 123 control children	Pooled swabs of tongue, palate, mucosa, and saliva	*Bulleidia* *Gemella* *Granulicatella*, *Rothia Schaalia* *Streptococcus*	*Neisseria* and *Haemophilus* ART initiation <90 days of life: *Corynebacterium* *Haemophilus* *Leptotrichia*

Only studies that presented a control group for comparison were included in this table.

Studies with less than 10 subjects per group were excluded due to debatable significance of the results.

*: the comparative group was made of HIV-negative smokers.

**: the comparative group was made of HIV-infected women without periodontitis.

ART, antiretroviral therapy; HOMIM, Human Oral Microbe Identification Microarray; MSM, men who have sex with men; N/D, no data available.

In terms of bacterial species, oral samples of PLWH present significantly richer bacterial communities than those of controls. *Micrococcus* spp, species of the *Spirochaetaceae* family (*Spirochaeta* spp, *Treponema* spp, *T. amylovorum*, and *T. azotonutricum*), *Prevotella melaninogenica* and *Rothia mucilaginosa* have been found related to HIV infection. Additionally, a significant reduction of viridans streptococci group, *Helicobacter* spp, and *Streptococcus pneumoniae* have been found associated to HIV infection (Hegde et al., 2014; Lewy et al., 2019; Perez Rosero et al., 2021).

The main findings regarding bacterial genera and species found in PLWH by comparison to controls are shown in **Table 2**.

### 4.3 Association between oral microbiota and periodontitis in HIV infection

**Figure 2:**
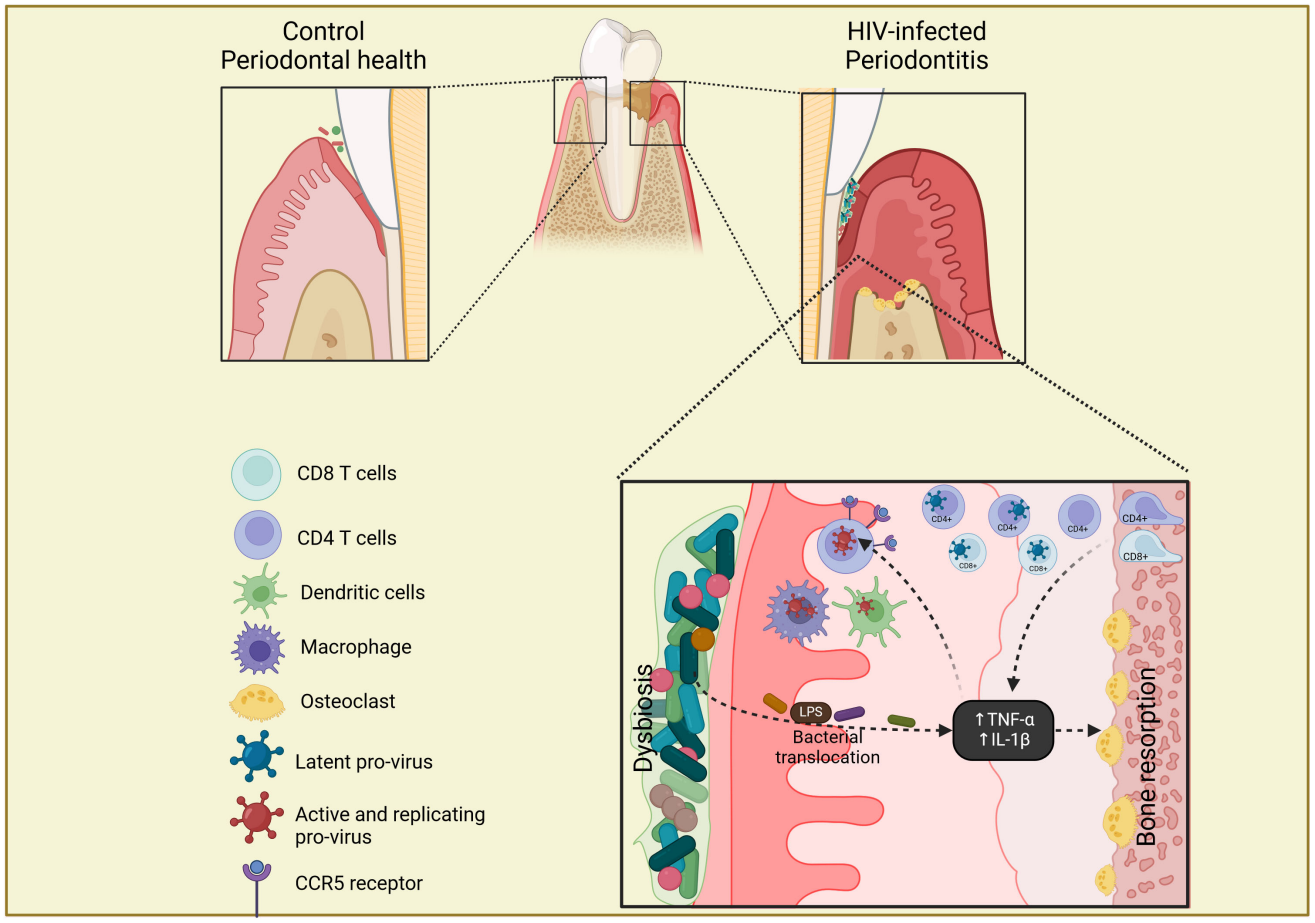
Scheme of gingival tissue as a potential reservoir of HIV. In well-controlled HIV-infected patients with periodontitis, the presence of chronic inflammation in the gingival tissue leads to inflammatory infiltrate, with the migration of CD4+ and CD8+ T cells with latent HIV integrated provirus, from the lamina propria of alveolar bone. The bacterial dysbiosis and translocation present in periodontitis increase the secretion of pro-inflammatory cytokines (TNF-α and IL-1β) that induce bone resorption, replication and reactivation of HIV provirus and up-regulation of CCR5 receptor on CD4+ T cells, macrophages and dendritic cells, which in turn increases the periodontal destruction. The Figure was created with BioRender.com.

Periodontitis has a great impact on the oral microbiota and the impact of HIV infection in periodontitis evolution has been approached by different studies. The pathogenesis of periodontitis in PLWH can be described as a reciprocal reinforcement of the two conditions, where the local dysbiosis present in the periodontal pocket leads to inflammation, bacterial translocation and destruction of the supporting tissues, which in turn enhances a pathogenic environment that perpetuates the periodontitis cycle (Hajishengallis & Chavakis, 2021). This cycle, illustrated in **Figure 2**, can be enhanced by the presence of chronic immunosuppression produced by HIV infection, leading to an increased destructive potential of periodontal tissues in these individuals (Aas et al., 2007; Annavajhala et al., 2020).

PLWH with severe forms of periodontitis were shown to present higher abundance of *Prevotella melaninogenica*, *Rothia mucilaginosa*, *Rothia dentocariosa*, and *Fusobacterium* compared to individuals with non/mild periodontitis. These patients were also found to exhibit a reduced abundance of *Streptococcus*, *Actinomyces* and *Granulicatella* compared to non/mild periodontitis in the subgingival biofilm (Annavajhala et al., 2020). Additionally, different stages of periodontitis present subtle microbial signatures in oral microbiota of PLWH, including the enrichment of the genera *Abiotrophia*, *Neisseria*, and *Kingella* regardless of the periodontal status (Noguera-Julian et al., 2017). In another study, *Haemophilus parainfluenzae* was shown to be significantly associated with PLWH, whilst *Streptococcus mitis* was most significantly associated with controls. Interestingly, these differences in the abundance of the different genera were not observed in periodontal healthy sites, where the overall bacterial composition tends to be more similar between PLWH and controls with only minor differences (Kistler et al., 2015).

In subgingival biofilm samples, differences of diversity have been reported in PLWH and controls with periodontitis, with higher alpha diversity along with lower beta diversity among PLWH (Annavajhala et al., 2020; Ramos Peña et al., 2022). Additionally, changes after the periodontal treatment were also pointed out for both groups, with a higher impact on alpha diversity reduction in PLWH with periodontitis undergoing non-surgical periodontal treatment (Ramos Peña et al., 2022). The lower abundance of classically periodontitis-related pathogens in PLWH has been associated to an increased pathogenic activity of commensal and health-related bacteria when in presence of HIV infection (Aas et al., 2007; Gonçalves et al., 2007; Guo et al., 2021b).

### 4.4 Association of oral microbiota to other clinical variables in periodontitis genesis

Other variables that have been analyzed for changes in the oral microbiota include perinatally infection/exposure, smoking, and pulmonary involvement.

A study that compared the oral microbiota of perinatally HIV-infected (PHIV) and HIV-exposed uninfected (PHEU) youth found an association between PHEU and periodontitis-associated bacteria. On the other hand, the PHIV patients presented lower abundance of *Corynebacterium*, a health-related species (Starr et al., 2018).

Smoking is a clinical variable that directly affects the oral microbiota and has been widely studied for its influence in several oral diseases, periodontitis and even oral cancer (Ahmed et al., 2021). When comparing HIV-infected smokers and non-smokers, bacteria in HIV-infected smokers were shown to present reduced alpha diversity in saliva and more richness in oral rinses. The genera *Granulicatella*, *Lactobacillus*, *Veillonella*, *Enhydrobacter*, *Streptococcus* and *Lactobacillus* were significantly increased in HIV-infected smokers, while *Neisseria*, *Prevotella*, *Pasteurella* and *Fusobacterium* were found to be significantly decreased in this group. The ratio of the abundance of phyla *Fusobacteriota:Proteobacteriota* and *Bacteroidota:Proteobacteriota* was significantly decreased in HIV-infected non-smokers compared to HIV-infected smokers (Mukherjee et al., 2018; Yang et al., 2020).

Due to the close anatomical relationship between the mouth, the nasal cavity and the lungs, it is reasonable to search for associations in the microbiota of these three sites. In HIV-infected patients with abnormal lung function (long term ART and normal CD4+ T cell count), alterations in the oral microbial communities have been found, with increased abundance of *Veillonella*, *Streptococcus* and *Lactobacillus*, while *Neisseria* was significantly decreased; in contrast, HIV-negative individuals did not present any association related to lung function. A reduced richness of the oral microbiota in HIV-infected patients with impaired capacity of the lungs for carbon monoxide was also observed. No difference regarding ART, CD4+ T cell count or viral load was found. The authors of this study suggest that the oral microbiota may serve as a biomarker of lung function in HIV-infected patients and may be associated to the pathogenesis of chronic obstructive pulmonary disease (Yang et al., 2020).

In another study that compared oral and airway microbiota in HIV-infected patients with pneumonia, a greater bacterial burden was detected in the oral *vs* airway microbiota in pneumonia; however, alpha diversity was similar across both microbiotas. Additionally, the respiratory pathogens *Streptococcus pneumoniae*, *Staphylococcus aureus*, *Haemophilus influenzae*, *Pseudomonas aeruginosa* and *Chlamydia pneumoniae* were present in the oral microbiota of HIV-infected patients. The clusters of both oral and airway microbiotas indicated a high similarity of the microbiota profiles, especially in ART-treated patients, suggesting an impact of ART in the oral and airway microbiota, and the presence of oral dysbiosis in HIV-infected patients with pneumonia (Iwai et al., 2012). The results of this study bring additional relevance to the necessity of prevention and treatment of periodontitis in HIV-infected patients.

### 4.5 Differences in the oral microbiota in relation to surrogate markers of HIV infection severity

#### 4.5.1 CD4+ T cell count

In PLWH, a relationship between chronic dysbiosis, aging, viral load, CD4+ T cell count and long-term ART has been suggested (Lewy et al., 2019). Low CD4+ T cell count has been associated with low relative abundance of *Haemophilus*, *Actinomyces*, *Ruminococcus*, *Rothia*, *Micrococcus*, *Acinetobacter* and *Klebsiella*; high CD4+ T cell count has been correlated with increased abundance of *Streptococcus* and *Lactobacillus* in the salivary microbiota, leading to a more balanced microbiota (Hegde et al., 2014; Lewy et al., 2019; Li et al., 2021). However, after an increase of the CD4+ T cell count over 300/mm^3^, the microbiota becomes more similar to HIV-negative controls (Hegde et al., 2014), suggesting that the immunosuppression caused by the depletion of CD4+ T cells in PLWH is related to the development of oral dysbiosis. Individuals with persistent low CD4+ T cell count after ART present significantly higher bacterial richness and alpha diversity compared to those with high CD4+ T cell count (>200/mm^3^) (Presti et al., 2018). Additionally, bacterial alpha diversity has been associated with serum levels of the inflammatory marker sCD14 (normal values 1.75-2.90 µg/ml): in case of high serum sCD14, enrichment of *Streptococcus* has been found, whist in case of low serum sCD14, enrichment of *Neisseria* and *Rothia mucilaginosa* has been found (Annavajhala et al., 2020).

Another interesting association observed in saliva of PLWH, both ART-treated and ART-naïve, is the reduction of oral neutrophils, the most abundant leukocytes in the oral cavity, related to low CD4+ T cell count and high bacterial diversity and richness. This might have an influence in the predisposition of PLWH to oral opportunistic infections, since neutrophils have an important role in the immune homeostasis of the oral cavity (Perez Rosero et al., 2021).

#### 4.5.2 Viral load in serum

The serum HIV load also seems to impact the oral microbiota since detectable viral load has been associated to increased diversity, while undetectable viral load has been associated to a decrease in alpha diversity and periodontitis-related bacteria, leading to a microbiota more similar to the one found in HIV-negative individuals (Ramos Peña et al., 2022). High HIV load has been correlated with the presence of *Prevotella* and *Veillonella* and negatively correlated with *Streptococcus* and *Lactobacillus*, which are two potentially beneficial genera (Lewy et al., 2019; Ramos Peña et al., 2022). Low viral load has been associated with reduced detectable bacterial taxa after 24 weeks of ART, while bacterial diversity remained similar in low and high viral load (>100,000 copies/ml). *Porphyromonas* has been found abundant in patients at baseline, and almost entirely absent after 24 weeks of ART, while *Treponema lecithinolyticum* was abundant even after 24 weeks under ART, both bacteria being of great periodontitis potential (Presti et al., 2018).

### 4.6 Differences in the oral microbiota in relation to ART

It is known that ART is able to restore the CD4+ T cell count and suppress the HIV viremia in most PLWH; however, several manifestations of the HIV infection can still appear in these patients, especially signs related to residual inflammation, affecting the gut and oral microbiotas, contributing to dysbiosis and to the pathogenesis of related diseases (Coker et al., 2021).

The effects of ART on the oral microbiota have been considered of small to modest magnitude, and similar to other clinical variables such as periodontal status, smoking and antibiotic usage (Griffen et al., 2019; Beall et al., 2023). Bacterial composition of PLWH under ART differs from that of PLWH without ART, and of controls. ART shifts the oral microbiota to be closer to that found in controls, although, even after long-term ART, the microbiota does not reach a composition equal to that found in controls. This diversification has also been seen in the esophageal microbiota of PLWH (Saxena et al., 2016).

The comparison of the oral microbiota in PLWH and controls shows that HIV infection has a significant impact on the oral microbiota; ART produces significant changes in the core oral microbiota, with increased abundance of *Neisseria* and *Haemophilus* and enrichment of LPS-secreting bacteria in periodontitis individuals. However, even in presence of effective ART, the association of HIV infection and periodontitis continues to alter the oral microbiota (Lewy et al., 2019; Perez Rosero et al., 2021; Beall et al., 2023).

In HIV-infected untreated patients, mean alpha diversity is more variable between individuals, with high abundance of *Bacteroidota*, *Bacillota* and *Proteobacteriota*. After 24 weeks of effective ART, the dominant phyla remain similar to baseline, without differences in beta or alpha diversity; however, bacterial communities became more similar between individuals, with enrichment of health-related genera such as *Fusobacterium*, *Campylobacter*, *Prevotella*, *Capnocytophaga*, *Selenomonas*, *Actinomyces*, *Granulicatella* and *Atopobium*, and decrease of pathogenic genera such as *Porphyromonas* and *Aggregatibacter* (Yang et al., 2020; Li et al., 2021; Cao et al., 2022). The bacterial communities within each individual present changes before and after ART, suggesting high person-to-person variability on salivary bacterial microbiota between baseline and 24 weeks after ART (Presti et al., 2018; Cao et al., 2022) (**Figure 1**).

While great impact on the gut microbiota has been described in PLWH under ART, especially when NRTI are used, this same impact has not been found on the oral microbiota. Likewise, ART increases the gut beta diversity of HIV-infected patients, but not the salivary beta diversity, when comparing PLWH to controls (Imahashi et al., 2021). Additionally, long-term ART has been associated to a decrease in the severity of periodontitis in PLWH (Gonçalves et al., 2007).

In addition, when comparing ART immunological responders to non-responders, taxonomic differences were noted, with higher abundance of the genus *Selenomonas* in the non-responder group, and higher abundances of *Cd* Saccharimonas and *norank_p_Saccharimonas* in the immunological responders; however, the overall microbiota structure was similar between both groups (Xie et al., 2021).

In patients exhibiting pneumonia, clusters of both oral and airway microbiotas were closely together, indicating a high similarity of the microbiota profiles, especially in PHLW under ART, suggesting similar impact of ART on the oral and airway microbiotas (Iwai et al., 2012).

### 4.7 Differences in the oral microbiota in relation to age

Aging is an important variable to be taken into consideration for the oral microbiota analysis. Several alterations take place in the mouth during the human development at both extremities of life, notably with changes in the dentition and diet in young children, and senescence-related alterations in elderly, including the occurrence of systemic diseases as diabetes *mellitus*, Sjögren’s syndrome, cardiovascular diseases and neurodegenerative diseases, which results in an increase of the basal level of inflammation (Zapata & Quagliarello, 2015). In PLWH, the presence of chronic immunosuppression and ART are to be taken into account, in addition to the previously mentioned factors.

In adults, age has been associated with increased bacterial diversity in PLWH and controls; older HIV-chronically infected patients under long-term ART present reduced abundance of oral commensal bacteria leading to a microbiota more similar to that of HIV-negative adults over time (Lewy et al., 2019).

Supragingival biofilm samples of HIV-infected children and teenagers under ART show similar microbial diversity than in HIV-negative individuals. Indeed, ART well-controlled HIV-infected children and teenagers presented similar oral microbiota to HIV-negative counterparts (Goldberg et al., 2015). However, another study, also using samples of supragingival and subgingival biofilms from HIV-infected children and teenagers presented opposed results, with more complex bacterial communities, increased richness and genera abundances. High abundance of *Bacillota*, *Streptococcus* spp, *Veillonella* spp and *Prevotella* spp and low abundance of *Fusobacteriota* and *Proteobacteriota* were found in PLWH but not in controls. While these differences could be attributed to several clinical variables related to the dental biofilm samples, the differences found in tongue biofilm samples were attributed exclusively to the presence of HIV infection (Gonçalves et al., 2019).

Young HIV-infected children (mean age of 4 years) present significantly lower diversity of bacterial species in saliva, with high mean count of *Fusobacterium periodonticum*, a normal oral species that act as a bridge between colonizers in the dental plaque. In this population, high viral load has been associated with *Gemella morbilorum*, *Staphylococcus intermedius*, *Streptococcus anginosus*, *Treponema denticola* and *Enterococcus faecalis*; however, no significant differences have been found between untreated patients and those under ART. Furthermore, the presence of concomitant infections, such as angular cheilitis and oral herpes, has been related to high levels of *Veillonella parvula*. When gingivitis is present, high prevalence of *Tannerella forsythia*, *Eikenella corrodens* and *Propionibacterium acnes* have been found in the saliva of HIV-infected patients, contributing to the establishment of altered microbiota since early age (Silva-Boghossian et al., 2008).

In children less than 6 years of age, a low CD4+ T cell count exerts a strong impact on the oral microbiota. Perinatal exposure is considered as a transient effect on the oral microbiota; indeed, perinatally HIV-exposed children initially present altered bacterial oral microbiota, but their microbiome tends to become more similar to that of non-HIV-exposed/HIV-negative children overtime (Coker et al., 2020).

When comparing the time of initiation of ART (before or after 6 months of age) in children (median age 11 years), the HIV-infected patients presented high abundance of *Granulicatella*, *Streptococcus*, *Gemella* and *Bulledia*, while *Neisseria* and *Haemophilus* were less abundant, independently of viral load and CD4+ T cell count. Earlier ART initiation was not associated to changes in the oral microbiota; however, the type of ART regimen did present associations with shifts in the genus-level abundances, especially lopinavir/ritonavir regimens, leading to a less diverse oral microbiota. Children receiving efavirenz-based regimens presented similar alpha diversity to HIV-negative individuals. Additionally, *Corynebacterium* has been found more abundant in immunosuppressed children (Kuhn et al., 2023).

According to the studies presented in this section, it can be concluded that in young people the alterations in the oral microbiota are mostly linked to the individual development, with a high impact of ART, whereas in the adult age the oral dysbiosis is strongly influenced by other clinical variables, such as smoking, periodontitis and changes in ART regimens.

## 5 Assessment of the mycobiome

### 5.1 Whole oral mycobiome

In HIV-negative healthy individuals, the interactions between bacteria and fungi are considered limited, notably in terms of alpha and beta diversity. While the bacteriome is strongly shaped according to age and sex, these factors have no influence on the mycobiome (Cheung et al., 2022). Nonetheless, with the introduction of immunosuppression and bacterial dysbiosis, alteration in the mycobiome can be expected in PLWH.

From 2007 to 2023, six studies that evaluated the oral mycobiome in PLWH and corresponding to the inclusion criteria defined above were selected and analyzed. A summary of each of these studies is presented in **Table 3**. The main mycobial genera/species found in PLWH in comparison to controls are shown in bold characters in **Table 2**.

**Table 3:** Presentation of the 6 studies selected for the analysis of the oral mycobiome in people living with HIV (PLWH).

	Reference	Population characteristics	ART status	Immune/viral status in HIV-infected patients	Type of sampling	Method of assessment	Main mycobial findings
1	Aas et al., 2007	14 HIV-infected adult males: - 5 with gingivitis - 8 with periodontitis - 1 with linear gingival erythema	NA	10 with CD4+ T cell count >300/mm^3^ and viral load <2,000 cp/ml 4 with CD4++ T cell count <200/mm^3^ and viral load >20,000 cp/ml	Subgingival biofilm	18S rDNA gene amplification and sequencing	*Saccharomyces cerevisiae* was the only fungal species detected in the linear gingival erythema subject and in periodontitis subjects with high viral loads. In periodontitis patients with low viral loads, *Candida albicans* was predominant, while *S. cerevisiae* was only a minor component.
2	Mukherjee et al., 2014	12 HIV-infected adults 12 control adults	8 PLWH under ART	11 with CD4+ T cell count <200 /mm^3^ and detectable viral load 1 with CD4+ T cell count >200 /mm^3^ and detectable viral load	Oral rinse	Culture on Sabouraud dextrose agar medium	The oral mycobiome of PLWH differs from that of controls. Decrease in abundance of *Pichia* (a resident oral fungus) in controls coincided with increase in abundance of *Candida* spp, which suggests an antagonistic relationship.
3	Mukherjee et al., 2018	48 HIV-infected smoker adults 24 HIV-infected non-smokers adults 24 control smoker adults	72 PLWH under ART	Mean CD4+ T cell count: - 838.87/mm^3^ in HIV-infected smokers - 808.83 /mm^3^ in HIV-infected non-smokers Viral load NA	Oral rinse	Internal transcribed spacer 1 (ITS1) sequencing	Fungal phyla did not differ significantly between the three cohorts. There was no difference in richness of the mycobiome at any taxon level. Diversity of mycobiome in HIV-infected non-smokers was significantly lower than that of HIV-infected or control smokers at phylum level, but not at genus level. Levels of *Candida* spp were increased in both HIV-infected and control smoker groups compared to the HIV-infected non-smoker group. *C. rugosa* was detected only in the control smoker group. Inter-kingdom correlations between the pathogenic genera *Candida* and *Neisseria* were negative in HIV-infected smokers but positive in HIV-infected non-smokers. These two genera may interact and influence microbial dysbiosis in the oral cavity.
4	Annavajhala et al., 2020	52 HIV-infected post-menopausal women with periodontitis: - none/mild: 4 - moderate: 16 - severe: 22	52 HIV-infected women under ART	43 with CD4+ T cell count >200 /mm^3^ and undetectable viral load 1 with CD4+ T cell count >200 /mm^3^ and detectable viral load	Saliva Subgingival biofilm Stools	Internal transcribed spacer (ITS) sequencing	Fungal alpha diversity was reduced in biofilm from teeth with higher clinical attachment loss and in saliva and biofilm from patients with a history of AIDS. Saliva samples from patients with severe periodontitis compared to those with no or mild periodontitis were significantly enriched in *C. albicans* and *C. parapsilosis.* Fungal oral mycobiome communities likely play a role in chronic systemic immune activation in PLWH.
5	Fidel et al., 2021	149 HIV-infected adults 88 control adults	149 PLWH under ART	CD4+ T cell count range: 1-1661 cells/mm^3^ Viral load range: 19–1.74 × 10^6^ cp/ml	Oral rinse	Fungal internal transcribed spacer 2 (ITS2) region amplification and sequencing	Specific interactions between fungi and bacteria often showed *Candida* species positively correlated with *Baccilota* or *Actinobacteria* and negatively correlated with *Fusobacteria, Proteobacteria* and *Bacteroidete*s. The fungal species per individual were low in number (average of 12) and largely dominated by 1 to 3 primary species in greatest abundance. Major clusters of fungal communities were identified, with the predominant species being *Saccharomyces cerevisiae, Candida albicans, Candida dubliniensis*, and *Malassezia restricta.* Although samples from the four major clusters were found in both the PLWH and control groups, a statistically higher association of the *C. dubliniensis* with the control group was found. The oral mycobiome, while diverse, is often dominated by a limited number of species per individual; it is affected by several clinical variables, including HIV positivity and ART, and shows genera-specific associations with bacterial groups. The impact of HIV/ART was much less than expected
6	Beall et al., 2023	257 HIV-infected adults 93 control HIV-high risk adults	71 PLWH w/o ART	Mean CD4+ T cell count: 426 /mm^3^ Mean viral load: 43,685 cp/ml	Oral rinse, before and after treatment for those under ART	V1-V3 16S amplification and sequencing	HIV was considered the primary influence on the community variance (2.7%) when comparing PLWH pre-ART and HIV-high risk subjects. No other clinical variables were significant contributors.

ART, antiretroviral therapy; cps/ml, copies per ml; NA, not available; PLWH, people living with HIV; w/o, without.

By contrast to the oral bacteriome, the composition of the fungal mycobiome is strongly influenced by HIV infection, while other clinical variables such as periodontitis, smoking, age and antibiotic usage have no significant influence on it (Beall et al., 2023). The smaller number of variables influencing the fungal communities by comparison to bacterial communities may be related to the relative scarcity of fungal species identified in the oral cavity (Fidel et al., 2021).

Contrary to bacterial diversity, fungal diversity has not been associated to soluble or cellular biomarkers of immune stimulation or T cell dysfunction (Annavajhala et al., 2020). Fungal alpha diversity has been found reduced in the subgingival biofilm and saliva of patients with AIDS history. While periodontitis classification has not been associated with changes in mycobial diversity, *Candida albicans* and *C. parapsilosis* have been found enriched in non/mild periodontitis individuals, and lower abundance of *Candida dubliniensis*, *Exserohilum turcicum*, *Guehomyces pullalans*, and *Debraryomyces hansenii*. On the other hand, severe periodontitis has been associated to decreased abundance of *Saccharomyces cerevisiae*, *Exseohilum, Guehomyces*, and *Debaryomyces* in both subgingival biofilm and saliva (Annavajhala et al., 2020).

*Candida* spp and *Penicillium* spp are two genera found in PLWH and controls, however, with different abundances. In PLWH, *Candida* spp has been found in lower abundance with a parallel higher abundance of *Pichia* spp, while the inverse was observed in controls (Mukherjee et al., 2014). While *C. dubliniensis* has been found predominant in controls, HIV-infected samples are shifted toward the *C. albicans* and *Malassezia restricta* predominant clusters (Fidel et al., 2021). These findings show that the oral mycobiome of PLWH differs from that of controls: while the *Candida* genus is present in both groups, the abundance of specific species differs between groups.

When smoking is considered for the mycobial analysis, differences between HIV-infected smokers *vs* HIV-infected non-smokers and HIV-negative smokers has been reported. *Candida* spp levels are increased in HIV-infected and HIV-negative smokers. Fungal diversity has been described as increased in HIV-infected smokers compared to HIV-infected non-smokers, suggesting that smoking does induce changes in the oral mycobiome (Mukherjee et al., 2018). Although the clinical implications of these findings are still not well understood, the effects of smoking, high levels of *Candida* spp and chronic immunosuppression could influence the prevalence of oral candidiasis in PLWH.

### 5.2 Oral *Candida* spp infection

Whereas the number of studies evaluating the whole oral mycobiome is limited in PLWH, oral candidiasis is considered the most common opportunistic infection in this population. Indeed, as pointed out in the previous section, *Candida spp* are the most frequent species colonizing the saliva of human individuals, with *C. albicans* as the most common species found in HIV-infected patients. The detection of high amounts of this species is associated with low CD4+ T cell count and use of long-term antibiotics, while high CD4+ T cell count and ART treatment are associated with decrease levels of *C. albicans* (Merenstein et al., 2013; dos Santos Abrantes et al., 2014; Jiang et al., 2014).

In PLWH, *Candida* spp colonization is the most important predictive factor for the development of oral candidiasis. Indeed, while in healthy individuals the presence of *C. albicans* in the oral microbiota represents a true commensalism, it is associated in PLWH to a higher expression of virulence factors, such as phospholipase and DNAse, that favor the evolution to candidiasis (de Paula Menezes et al., 2016). Although ART treatment is able to decrease the expression of virulence factors and the carriage rate, the latter remains higher than that found in HIV-negative controls (Jiang et al., 2014; de Paula Menezes et al., 2016). It was also shown that the ART treatment was able to correct the dysfunctions in the production of Th1/Th2/Th17 cytokines and human beta defensin 2 in HIV-infected patients with oral candidiasis (Yong et al., 2018).

Other factors that influence *Candida* spp carriage in PLWH are the occurrence of periodontitis in adults (Lourenço et al., 2017; Lomeli-Martinez et al., 2019), and the presence of caries in children (das Chagas et al., 2009); by contrast, carriage levels were decreased in periodontally healthy individuals and after treatment of children with caries. A mutualism relationship between periodontitis, caries and oral candidiasis has been found in healthy individuals (Hong et al., 2020); it could be valuable to investigate this association in PLWH.

In addition, the growing resistance to antifungal drugs that affects *Candida* strains in some HIV populations should be considered while studying the prevention and treatment of oral candidiasis (dos Santos Abrantes et al., 2014).

## 6 Assessment f the oral virome and of viral coinfections

### 6.1 Overall oral virome

There are few studies documenting the oral virome of HIV-negative individuals, with or without periodontitis. In 2016, Monaco et al. investigated a cohort of HIV-negative and HIV-infected subjects and those either treated with ART or untreated. Low peripheral CD4+ T cell counts were associated with an expansion of enteric adenovirus sequences and this increase was independent of ART treatment (Monaco et al., 2016). Girija & Ganesh (2022) reported a predominance of phages belonging to the *Siphoviridae* and *Myoviridae* families and a high abundance of streptococcus-related phages. Eukaryotic DNA viruses were dominated by members of the *Herpesviridae* family (Girija & Ganesh, 2022). A study developed in 20 HIV-infected men who have sex with men (MSM) exhibiting different stages of HIV infection, with 5 HIV-negative MSM as controls, three phage families and a eukaryotic DNA family were found to be the more abundant, regardless of HIV status, namely *Siphoviridae*, *Herpesviridae*, *Myoviridae*, and *Podoviridae*, respectively. Interestingly, the *Lymphocryptovirus* genus of *Herpesviridae*, to which belong human herpesvirus 8 (HHV-8) associated to Kaposi sarcoma (KS), was particularly rare in HIV-negative controls, but abundant in HIV-infected patients (Guo et al., 2021a). Another study compared the oral and anal viromes of 78 MSM and transgender women, with and without HIV infection; human papillomaviruses (HPV) were predominant in the anal area whereas phages and *Herpesviridae* (notably HHV-7 and HHV-8) predominated in the oral site, whatever the groups (Lacunza et al., 2023).

### 6.2 Specific viruses involved in oral lesions during HIV infection

HIV infection commonly leads to the development of oral lesions related to reactivation of quiescent DNA viruses, including mainly members of the *Herpesviridae* family and different oncogenic types of the *Papillomaviridae* family (HPV) (Anonymous. EC-Clearinghouse on Oral Problems Related to HIV Infection and WHO Collaborating Centre on Oral Manifestations of the Immunodeficiency Virus, 1993). These viral infections in PLWH are highly correlated to their immune status as evaluated by the CD4+ T cell count and viral load, with a high increase of their frequency in case of severe immunosuppression.

#### 6.2.1 Herpesviruses

Both types 1 and 2 of herpes simplex virus (HSV) are frequently found in the oral sphere of healthy subjects. HSV infection is considered a frequent viral infection in PLWH and is responsible for severe complications in case of immunosuppression (Grando et al., 2005; Yunusa et al., 2019). From studies relative to healthy individuals, associations between HSV-1 and Epstein-Barr virus (EBV) infections, and progression of periodontitis have been reported (Das et al., 2012). Considering the high prevalence of both infections in PLWH, their role on periodontitis progression can be expected although not already reported.

Cytomegalovirus (CMV) is frequently present in immunocompetent patients but is responsible for severe infections when it reactivates in immunosuppressed patients, notably at the late stages of HIV infection; it has been frequently reported in oral lesion of PLWH, possibly in coinfection with HSV and EBV (Grando et al., 2005). The presence of CMV co-infection in the saliva and periodontal pockets of periodontitis PWLH had been carried out, however with conflicting results (Grande et al., 2011; Fonseca et al., 2018).

Epstein-Barr virus (EBV) has been associated to the development of premalignant and malignant lymphoproliferative diseases in the general population, notably in the naso-pharyngeal and oral areas (de Lima et al., 2019). In PLWH, EBV is associated with oral hairy leukoplakia, which constitutes an immunosuppression marker for severe HIV infection. Increased EBV viral load in saliva has been related to HIV-infection with low CD4+ T cell count (Rosseto et al., 2023). EBV has been detected in cytology samples of the oral mucosa in a significantly higher proportion in PLWH than in controls (42.1% *vs* 16.6%), notably in the lateral border of the tongue and in oral mucosa (Ammatuna et al., 2001). Interestingly, EBV has been detected in the periodontal pocket in a high percentage (85.6%) of PLWH, with a linear correlation between HIV and EBV viral loads (Grande et al., 2011; Vincent-Bugnas et al., 2013). In another study, periodontitis has been linked to high EBV infection in the periodontium of controls, whereas, in PLWH, EBV viral load has been found high independently of the periodontal status, suggesting an association with HIV infection (Jácome-Santos et al., 2020).

KS, the most common malignancy found in PLWH, is consecutive to the infection by HHV-8, a virus that is latent in oral mucosal cells (Lacunza et al., 2023). Significant alterations in the oral microbiota are observed in PLWH exhibiting HHV-8-associated oral KS, including saliva dysbiosis and a marked diminution of bacterial alpha diversity and richness when compared to PLWH without KS. These changes can be related to advanced immunodeficiency but also to the presence of the HHV-8 infection (Gruffaz et al., 2020). The oral dysbiosis consecutive to HHV-8-associated KS includes an increased abundance of *Streptococcus* species and of *S. aureus*, which can contribute to the reactivation of latent virus from infected oral cells, facilitating the dissemination of HHV-8 in the oral cavity and promoting the initiation and progression of the malignancy (Dai et al., 2022).

#### 6.2.2 Human papillomavirus

HPV infection is considered the most common sexually-transmitted disease in humans. It has a frequency of approximately 80% among PLWH, especially for the HPV-16 and HPV-18 that are the most frequent high-risk types responsible for cancers in humans (Anaya-Saavedra et al., 2013; Visalli et al., 2021). The increased frequency of oral lesions associated to both HPV low- and high-risk types has been related to the increase in life expectancy of PLWH under long-term ART. It is noticeable that high viral loads of HPV may be found even in patients that maintain undetectable HIV load and high CD4+ T cell count (Anaya-Saavedra et al., 2013; Camacho-Aguilar et al., 2018). HPV DNA in saliva of PLWH has been more frequently detected than in controls (33% *vs* 7.14%, respectively), with an incidence three times higher in PLWH, especially in high-risk sexual behaviour practitioners (Giuliani et al., 2020; Visalli et al., 2021). Moreover, in PLWH, HPV coinfection has been found prevalent in EBV-associated oral hairy leukoplakia and also in the surrounding oral mucosa where no malignant lesion is present (Ammatuna et al., 2001; Correnti et al., 2010).

## 7 Discussion

In this section, we will first recapitulate how oral immune dysfunction and mucosal immune cells may be associated to some of the microbiome changes and pathology. Then, we will summarize the major findings established through this extensive analysis of the literature concerning the characteristics of the different bacterial, fungal and viral microbiotas found at the oral level in PLWH by comparison to those of controls, with a special attention to the links with persistent periodontitis.

As recalled in the introductory section of this review, the impact of HIV infection on gut microbiota is clearly established: HIV infection creates a disruption of the intestinal immune barrier which leads to the translocation of microbial products inducing an immune hyperactivation. The exhaustion of CD4+ T cells occurs rapidly, especially the Th17 subset that produces IL-17; this cytokine plays a key role in mucosal integrity by controlling the secretion of antimicrobial peptides involved in the defence against bacterial infection and of pro-inflammatory cytokines associated to neutrophil activation. At the intestinal level, the failure of Th17 and Treg cells induces a loss of mucosal integrity with microbial translocation and persistent inflammation. ART is able to restore the CD4+ T cell count, but only partially the Th17 subset, which results in a persistence of inflammation at the intestinal level. If similar mechanisms of chronic inflammation are observed at the oral level in the course of HIV infection, the microbiome changes are less clear-cut and their role in the genesis of immune hyperactivation is more difficult to establish, both in human studies and in non-human SIV-primate models.

As a preliminary remark to the discussion of microbiota analyses, it must be stressed that it is difficult to compare the different studies that were analysed in this review due to their heterogeneity in terms of collected samples, technologies used for analysing the microbiotas, PLWH and controls that were chosen, clinical conditions of patients (including age, circumstances of HIV contamination, smoking, airway infections), presence or not of periodontitis, stage of HIV infection (including immune status and viral load), ART treatment with different regimens, parallel analysis of oral and gut microbiota, etc. This huge heterogeneity explains, in large part, the different and sometimes contradictory results that were reported in this review. Nevertheless, it can be possible to drive some general trends from this extensive analysis of the literature (see **Tables 1**–**3**).

Concerning the bacterial microbiota, the beta diversity of gut samples presents significant differences between PLWH and controls, pointing out an association between HIV infection and gut dysbiosis; however, these significant changes have not been found in the oral microbiota when the only variable taken into consideration was HIV infection. At the oral level, the main taxonomical differences found in PLWH have been related to increased abundance of genera such as *Campylobacter, Granulicatella, Neisseria, Rothia* and *Selenomonas*, and decreased abundance of *Actinomyces, Lactobacillus, Peptostreptococcus* and *Treponema* (**Table 2** and **Figure 1**).

With regard to periodontitis, its pathogenesis in PLWH can be described as a reciprocal reinforcement of the two conditions, where the local dysbiosis present in the periodontal pocket leads to inflammation, bacterial translocation and destruction of the supporting tissues, which in turn enhances a pathogenic environment that perpetuates the periodontitis cycle (**Figure 2**). HIV-infected patients with severe forms of periodontitis were shown to present higher abundance of *Prevotella melaninogenica*, *Rothia mucilaginosa*, *Rothia dentocariosa*, *Fusobacterium* and *Streptococcus* compared to individuals with non/mild periodontitis. Patients with severe periodontitis were also found to exhibit a reduced abundance of *Streptococcus*, *Actinomyces* and *Granulicatella* compared to non/mild periodontitis in the subgingival biofilm. Interestingly, these differences in the abundance of the different genera were not observed in periodontal healthy sites where the overall bacterial composition tends to be more similar to that of HIV-negative controls.

In PLWH, a relationship between chronic dysbiosis, high viral load, and low CD4+ T cell count has been suggested. Furthermore, after an increase of the CD4+ T cell count over 300/mm^3^, the microbiota becomes more similar to HIV-negative controls, suggesting that the immunosuppression caused by the depletion of CD4+ T cells in PLWH is related to the development of oral dysbiosis. The viral load also seems to impact the oral microbiota since high viral load has been associated to increased diversity, including notably the presence of *Prevotella* and *Veillonella*, while undetectable viral load has been associated to a decrease in alpha diversity and periodontitis-related bacteria, leading to a microbiota more similar to the one found in HIV-negative individuals.

While great impact on the gut microbiota has been described in PLWH under ART, especially when NRTI are used, this same impact has not been found on the oral microbiota. Indeed, the effects of ART on the oral microbiota have been considered of small to modest magnitude, and similar to other clinical variables such as periodontal status, smoking and antibiotic usage. Although under ART the oral microbiota trends to be closer to that found in controls, it does not reach an equal composition, even after long-term therapy. However, long-term ART has been associated to a decrease in the severity of periodontitis in PLWH.

In PLWH with pneumonia, the clusters of both oral and airway microbiotas indicated a high similarity, especially in ART-treated patients, suggesting an impact of ART on both microbiotas, and the presence of oral dysbiosis in HIV-infected patients with pneumonia. These results plead for the necessity of preventing and treating periodontitis in PLWH.

In terms of oral fungal microbiota, unlike the oral bacteria, HIV infection has been appointed as a significant variable for changes in the composition of the mycobiome, while other clinical variables such as periodontitis, smoking, age and antibiotic usage do not influence significantly the oral mycobiome. The smaller number of variables influencing the fungal communities than those that influence the bacterial ones may be related to the relatively small number of fungal species identified in the oral cavity. Smoking, high levels of *Candida* spp and chronic immunosuppression could influence significantly the prevalence of oral candidiasis in PLWH.

With regard to the oral virome, very few studies are available both in PLWH and controls. Besides phages that constitute the major part of the virome, members of the *Herpesviridae* family are the most abundant eukaryotic viruses found in the oral cavity. In HIV-infected patients, they can exert a major pathogenic role in case of severe immunosuppression, notably HSV, CMV, EBV and HHV-8. By contrast, oncogenic HPV were shown to be at the origin of potentially malignant and malignant lesions of the oral cavity even in PLWH under ART with controlled HIV infection, which justifies a careful long-term surveillance of these infections.

## 8 Future perspective

The understanding of the interactions between oral microbiota and immune functions in HIV-infected patients under ART has considerably progressed in the recent years but still needs significant improvement. Some of the points that would need to be clarified are listed below:

- in line with what is done for the intestinal microbiota, a standardisation of the samples and methods used for analysing oral microbiota is urgently needed in order to help the comparison of the results obtained through different studies;
- given the interactions between the different mucosal systems, it would be useful to conduct studies analysing in parallel the microbiotas of several anatomic sites, notably oral, intestinal and genital levels;
- the oral cavity is particularly prone to chronic inflammation linked to multifactorial diseases including caries, periodontal diseases, candidiasis, recurrent viral infections; while in healthy individuals the pathophysiology is more clearly understood within the recent literature; the role HIV infection in the oral microbiota needs further analysis to stablish the level of repercussion of chronic inflammation, immunosuppression, and ART treatment in oral infectious diseases;
- probiotics able to correct oral dysbiosis have been proposed to correct the change in oral microbiota, notably in the context of HIV infection (Cunningham-Rundles et al., 2011; Moyes et al., 2016); rigorous and independent evaluations of these approaches must be favoured to appreciate their ability to improve mouth health.

To close this review, we address the question of the potential role of oral microbiota and dysbiosis in the establishment of an HIV gingival reservoir. Considering the gathered data on the changes in the oral microbiota of HIV-infected patients, oral dysbiosis could be considered an effect of HIV infection. Additionally, oral dysbiosis has been highly related to the inflammatory process produced by periodontitis and long-term ART (Starr et al., 2018; Lewy et al., 2019; Annavajhala et al., 2020). A synergic relationship between HIV-infection and periodontitis has been hypothesized by our group (Jotwani et al., 2004; González et al., 2009; Polvora et al., 2018; Sereme et al., 2022) on the following arguments: (i) presence of altered microbial communities in saliva, oral mucosa and periodontal pocket at bacterial, fungal and viral levels, with translocation of pathogens and/or their subproducts into the mucosa; (ii) continuous recruitment of mononuclear infiltrates into the gingival tissue as a consequence of the inflammatory response to periodontitis (Hajishengallis and Chavakis, 2021), potentially exacerbated by the HIV-induced chronic immunosuppression; and (iii) integration of HIV DNA into the genome of lymphocytes, macrophages and dendritic cells present in the gingival tissue (Chen et al., 2022) that, in the context of chronic local inflammation, can change from a latent to an activated state, which is the definition of an HIV reservoir. Works are in progress to validate this hypothesis.

## 9 Conclusion

This review has highlighted the presence of an altered status of the oral microbial communities in PLWH. The results that we have summarized herein plead for the potential role of oral inflammatory diseases, and notably periodontitis, in triggering the delicate balance of HIV replication controlled by modern ART regimens. More studies approaching the implications of the oral microbiota dysbiosis in the pathogenesis of periodontitis in PLWH and the role of gingival tissue as a reservoir of latent HIV provirus are still needed.

## Author contributions

DRP: Data curation, Formal analysis, Investigation, Methodology, Validation, Writing – original draft. SP: Validation, Writing – review & editing. AGL: Validation, Writing – review & editing. BP: Conceptualization, Formal analysis, Supervision, Validation, Writing – review & editing. TB: Conceptualization, Funding acquisition, Supervision, Validation, Writing – review & editing. AM: Conceptualization, Funding acquisition, Methodology, Supervision, Validation, Writing – review & editing.
